# On the effect of network structure and synaptic mechanisms on sustained bursting activity

**DOI:** 10.1186/1471-2202-14-S1-P247

**Published:** 2013-07-08

**Authors:** Tuomo Mäki-Marttunen, Jugoslava Aćimović, Keijo Ruohonen, Marja-Leena Linne

**Affiliations:** 1 Department of Signal Processing, Tampere University of Technology, Tampere, 33101, Finland; 2 Department of Mathematics, Tampere University of Technology, Tampere, 33101, Finland

## 

The sustained activity in recurrent networks has been under wide computational examination in studies concerning, e.g., working memory and epilepsy. Synaptic and cellular mechanisms for sustained activity have been reviewed in [1], and the optimal structural features for sustained activity have been sought for in [2]. In this work, we analyze the effect of network structure and synaptic mechanisms on the sustained high-frequency network-wide activity, i.e., sustained network bursts. In more detail, we assess the degree to which the neuronal activity can be maintained by changing fine details of network structure, given a certain set of synaptic mechanisms (e.g., short-term plasticity).

The neurons in our study are modeled as point-neurons that are activated by noisy fluctuations of the membrane potential. Given strong enough recurrent excitatory connections, the spontaneous firing extends to a network-wide sustained activity. Experimental *in vitro *data (e.g., [3]) show that this emergent activity dies out and restarts spontaneously in dissociated cultures. Several mechanisms have been suggested for ceasing the sustained activity, namely, the delayed activation of the inhibitory population, depletion of glutamatergic resources, and synchronization of the excitatory population [1,4]. The main focus of this work lies on the latter two mechanisms. We employ the integrate-and-fire neuron model with short-term depression [4] for deriving the main results. We also use a more biophysically realistic integrate-and-fire model that combines different synaptic currents, e.g., AMPA and NMDA [5], and a yet more detailed Hodgkin-Huxley-based model [6] to confirm our results. We consider four essentially different classes of network structure: 1) an Erdős-Rényi type of random network 2) a locally connected network, 3) a random network with high occurrence of directed loops of length 6, and 4) a random network with high number of triples of nodes constituting a feed-forward loop. The in-degree distribution of all networks is kept fixed in order to ensure that the networks are comparable.

Our results reveal links between the network excitability and the network structure. In purely excitatory networks with short-term depression, the amount of activity is increased with the synaptic strength, first from spontaneous tonic firing to spontaneous network-wide bursting activity and finally to long or ceaseless bursts. We show that the range of values of synaptic strength for observing one of these three modes depends on the choice of network structure. The networks with a high number of feed-forward loops show an increased ability to cease the burst in the regime of high synaptic strength, whereas the networks with a high number of 6-loops require lower synaptic strength in order to express ceaseless bursting activity. We calculate the parameter ranges of the named three modes of activity for several variations of synaptic currents. We also study the effect of an inhibitory subpopulation on the three modes. The results are discussed in comparison to "superbursts" that can be observed in dissociated cultures [7]. Our results could help in identifying structures that promote sustained bursting activity and further the understanding of contribution of different synaptic mechanisms.
